# Rapid Synthesis of a Long Double-Stranded Oligonucleotide from a Single-Stranded Nucleotide Using Magnetic Beads and an Oligo Library

**DOI:** 10.1371/journal.pone.0149774

**Published:** 2016-03-01

**Authors:** Sumate Pengpumkiat, Myra Koesdjojo, Erik R. Rowley, Todd C. Mockler, Vincent T. Remcho

**Affiliations:** 1 Department of Chemistry, 153 Gilbert Hall, Oregon State University, Corvallis, Oregon, United States of America; 2 Department of Botany and Plant Pathology, Oregon State University, Corvallis, Oregon, United States of America; 3 Donald Danforth Plant Science Center, 975 North Warson Rd., St. Louis, Missouri, United States of America; Northeastern University, UNITED STATES

## Abstract

Chemical synthesis of oligonucleotides is a widely used tool in the field of biochemistry. Several methods for gene synthesis have been introduced in the growing area of genomics. In this paper, a novel method of constructing dsDNA is proposed. Short (28-mer) oligo fragments from a library were assembled through successive annealing and ligation processes, followed by PCR. First, two oligo fragments annealed to form a dsDNA molecule. The double-stranded oligo was immobilized onto magnetic beads (solid support) via streptavidin-biotin binding. Next, single-stranded oligo fragments were added successively through ligation to form the complete DNA molecule. The synthesized DNA was amplified through PCR and gel electrophoresis was used to characterize the product. Sanger sequencing showed that more than 97% of the nucleotides matched the expected sequence. Extending the length of the DNA molecule by adding single-stranded oligonucleotides from a basis set (library) via ligation enables a more convenient and rapid mechanism for the design and synthesis of oligonucleotides on the go. Coupled with an automated dispensing system and libraries of short oligo fragments, this novel DNA synthesis method would offer an efficient and cost-effective method for producing dsDNA.

## Introduction

Oligonucleotide synthesis, the chemical synthesis of nucleic acids, has become an important tool in the field of molecular biology. Synthetic oligonucleotides have been utilized in numerous applications such as diagnosis of genetic and infectious diseases, new drug discovery, and disease treatment. Oligonucleotide synthesis has a long history, starting with a synthetic approach developed by Marvin Caruthers in early 1980s[1]. Solid-phase phosphoramidite chemistry is a well- established 4-step process, which elongates a chain of nucleotide from the 3’end to the 5’end, and is used by many commercial DNA synthesizers. The phosphoramidite chemistry has enabled routine synthesis oligos up to 100 nt with error rates of 1 in 200 nt or better[2], yet provides short oligonucleotides owing to the fact that the method adds one base at a time to the growing oligonucleotide chain. Each step in the synthetic cycle must have very high yield in order to obtain a final product in the required amount with a very low accumulated error rate. For example, for 200 nt oligo synthesis, 99% yield from each cycle will result in 13% yield of the desired final product. The longer the desired oligomer, the lower they yield that can be obtained from the synthesis process.

To assemble longer DNA strands, a set of pre-synthesized oligonucleotides can be used as building blocks and assembled using enzymatic methods. Ligation-based assembly is a method to join overlapping oligonucleotides using DNA ligase to form a longer gene. Here, all of the oligos are mixed together with DNA ligase and are thermocycled for annealing and ligation to build the gene product. The Polymerase Chain Reaction (PCR) is then used to amplify the full-length product. The drawbacks of the method are a relatively high probability of generating ligation by-products and the need for multiple overlapping oligos along the entire length of both strands.

Most gene synthesis today is conducted by service companies, and typically employs one or more of four different general approaches: ligation[3,4], PCR-mediated assembly[5], convergent assembly[6], or solid-phase assembly[7–10]. These approaches are relatively simple and published protocols are available. Recently, technologies and applications of DNA synthesis have been reviewed[2,11,12]. However, while these approaches generally succeed in producing usable oligos, they each have limitations than can lead to errors in assembly[13]. Additionally, some sequences are impossible to synthesize using these approaches, and the methodologies, which rely on laboriously constructed large (up to 200-base) oligos, are time consuming and error-prone.

As implemented by service companies, gene synthesis is still being conducted via a large-scale, semi-automated process using custom-synthesized individual DNA oligonucleotides. Because every gene is different, each is synthesized anew for each individual purchase order. Attempts have been made to automate the gene synthesis approaches described above by using DNA microarrays or microfluidic devices[14,15] in synthesis platforms that use ligation or PCR-mediated assembly. However, these technologies and approaches required custom synthesis, purification and amplification of relatively long oligonucleotides that are then joined into longer DNA molecules. In the microarray format, these approaches also tend to produce DNA products of lower-than-ideal purity. Accumulated errors and truncated DNA products can be significant problems as there is no purification following each synthetic step[16]. This is exacerbated by decreasing product yields with longer oligonucleotides due to the fact that chains may either stop growing or incorporate undesired nucleotides. For DNA synthesis in microfluidic systems, the materials used for fabrication must have excellent chemical resistance given the variety of chemicals and organic solvents used in standard DNA synthesis [17].

In this paper, we demonstrated enzymatic DNA synthesis using magnetic beads as solid supports. Magnetic beads have been widely utilized as solid supports for biomolecule separation, as they facilitate washing and easy manipulation of the sample. Typically, streptavidin is coupled to magnetic beads to specifically capture a target of interest such as a nucleic acid, protein, or antibody that has been biotinylated. Recently, chemical gene synthesis using magnetic beads as the solid support was presented [6]. Short double stranded (ds) oligo fragments 8 bp long were assembled to form longer *32 bp intermediate single stranded fragments* through ligation. The target DNA sequence of 128 bp was constructed from four sets of intermediate fragments in a pair-wise manner.

**Fig 1:**
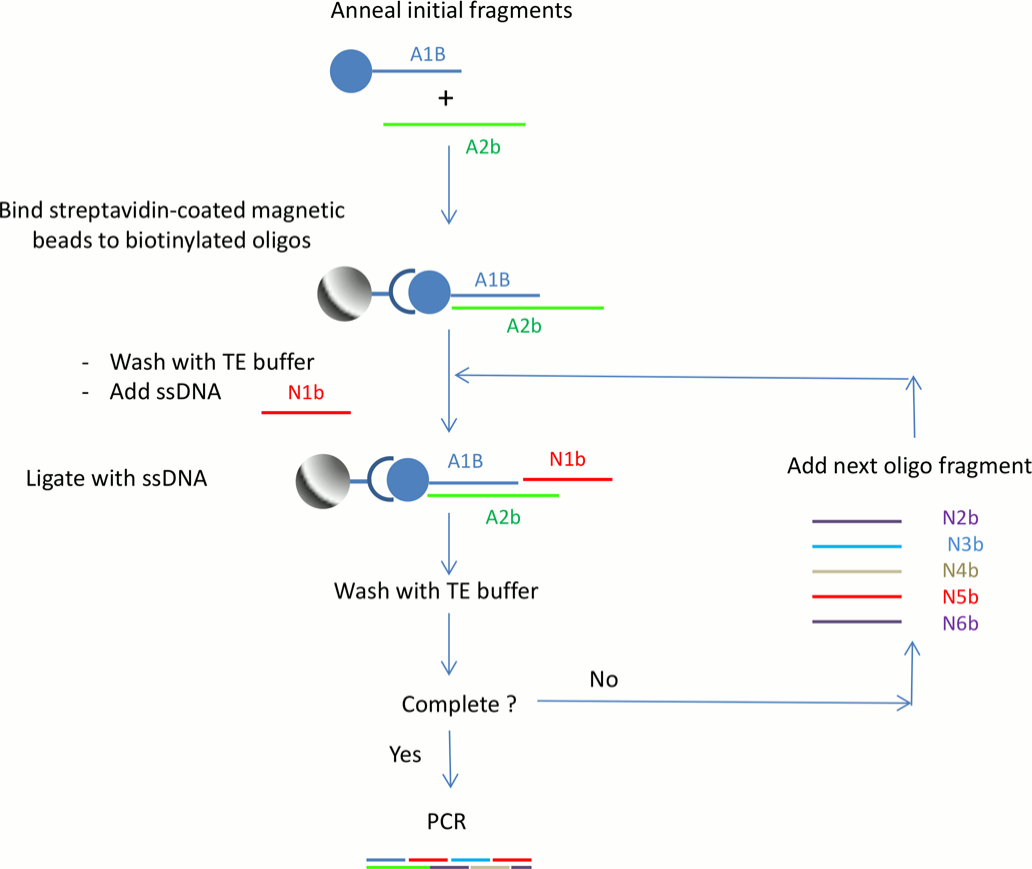
Schematic diagram for the proposed dsDNA synthesis. The overall procedure for dsDNA synthesis is composed of three processes: annealing, binding of streptavidin coated magnetic beads to biotinylated oligos, and ligation.

Herein, we describe a proof of principle study for the construction of a *full-length double-stranded DNA molecule from short fragments of single-stranded nucleotides* (ss-oligonucleotides). 28-mer oligo fragments were used as the building blocks to assemble the full length DNA. The gene was constructed by adding one ssDNA fragment at a time to extend the length of a target sequence through repeated ligation reactions. T4 DNA ligase was used to catalyze the formation of phosphodiester bonds between the adjacent 3’-hydroxy and 5’-phosphate termini in the double-stranded DNA. Streptavidin-coated magnetic beads were utilized as the solid supports for easy means of washing excess reagents at the completion of each ligation process. Without magnetic bead separation for each ligation step in the process, it is impossible to clean or eliminate the unreacted ssDNA from the enzymatic reaction. The magnetic bead purification approach provides each ligation reaction with only main chain DNA on the building block and the fragment ssDNA to be annealed and ligated to the main chain. Compared to conventional ligation-based assembly, our protocol generates more pure and accurate DNA final products, and affords an easy path to full automation through the use of automatic liquid dispenser, work that is ongoing in our laboratory. The overall procedure is shown in Fig 1. In this paper, we demonstrated the successful synthesis of a long dsDNA from a 28-mer library through successive ligation reactions. Combined with an automated system, this novel strategy will provide a powerful and rapid method for synthesizing custom dsDNA molecules in a standard laboratory.

## Materials and Methods

### Materials

**Fig 2:**
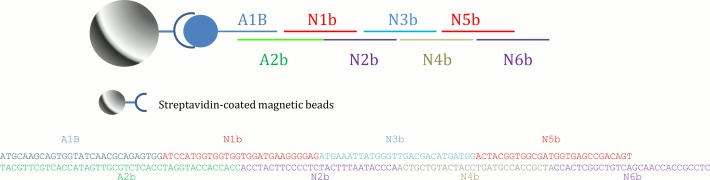
Schematic of the building blocks for DNA construction. Streptavidin-coated magnetic beads were used as solid support for dsDNA synthesis and oligo fragments were ligated to the building block one at a time.

All single-stranded oligonucleotides were purchased from Integrated DNA Technologies (Coralville, IA) with the 5’ ends of the oligos phosphorylated. A schematic of the building blocks for construction of the target DNA molecule is shown in Fig 2. The building blocks (N1b-N6b) were synthesized as 28 nucleotide-long fragments that overlapped the next fragment by 14 bases. The first two fragments (A1B and A2b) were designed to be 29 and 43 nucleotides long, respectively. All oligonucleotides were prepared at 100 μM (Table 1) and were stored at 4°C throughout the study. Streptavidin-coated magnetic beads, Dynabeads M-270, were obtained from Invitrogen™/Life Technologies (Grand Island, NY). Quick Ligation™ kits were obtained from New England Biolabs (Ipswich, MA).

**Table 1:** Sequence of the oligo fragments used to form the complete dsDNA.

Sequence name	Sequence	Number of nucleotides
Biotin-A1B	Biotin -5’-ATGCAAGCAGTGGTATCAACGCAGAGTGG-3’	29
A2b	3’-TACGTTCGTCACCATAGTTGCGTCTCACCTAGGTACCACCACC-5’	43
N1b	5’-ATCCATGGTGGTGGTGGATGAAGGGGAG-3’	28
N2b	3’-ACCTACTTCCCCTCTACTTTAATACCCA-5’	28
N3b	5’-ATGAAATTATGGGTTGACGACATGATGG-3’	28
N4b	3’-ACTGCTGTACTACCTGATGCCACCGCTA-5’	28
N5b	5’-ACTACGGTGGCGATGGTGAGCCGACAGT-3’	28
N6b	3’CCACTCGGCTGTCAGCAACCACCGCCTC-5’	28

Annealing buffer (10×) consisted of 100mM Tris-HCl, 500 mM NaCl, 10mM EDTA, was adjusted to pH 7.4 with NaOH. The ligation kit contains quick ligation buffer (2× QLB) (132 mM Tris-HCl, 20 mM MgCl_2_, 2mM dithiothreitol, 2mM ATP, 15% PEG6000, pH 7.6 at 25°C) and T4 ligase. EmeraldAmp® GT PCR Master Mix was obtained from Takara Bio Company (Mountain View, CA). DNA Ladder (O’RangeRuler 20 bp) was obtained from Life Technologies (Grand Island, NY).

Binding and washing buffer (2× B&W) buffer contains 10mM Tris-HCl, 1mM EDTA, 2M NaCl, and 0.05% Tween20, pH 7.5. Binding and washing 1× buffer (B&W) was prepared by diluting 2× B&W buffer with TE buffer in equal proportions. The TE buffer contains 10mM Tris-HCl, 1mM EDTA, and 0.05% Tween20.

Tris-acetate-EDTA (TAE) 50× buffer was obtained from Bio-Rad (Hercules, CA). It was diluted to 1× buffer with deionized water. The composition of 1× TAE buffer was 40mM Tris (pH 7.6), 20 mM acetic acid, and 1 mM EDTA.

### Methods

#### Preparation of Streptavidin-coated magnetic beads

50 μL of magnetic beads were washed with 50 μL of 2× B&W buffer (10mM Tris-HCl pH 7.5, 1mM EDTA, 2M NaCl, and 0.05% Tween20) for three times to remove excess sodium azide bacteriostatic agent and resuspended in 50 μL, 1X B&W buffer.

#### Annealing process

5 μL of stock solutions (100 μM) of the first (A1B) and second (A2b) single-stranded oligo fragments were mixed with 80 μL of deionized water and 10 μL of 10× annealing buffer (100mM Tris-HCl, 500 mM NaCl, 10mM EDTA, pH 7.4). The mixture was vortexed and heated in a water bath at 95°C for 10 min, and was slowly cooled to room temperature. The final concentration of each oligonucleotide was 5 μM.

#### Binding streptavidin coated-magnetic beads and biotinylated oligos

The annealed products from the first two fragments (Biotin A1B-A2b) were incubated with the magnetic beads by gentle-rotation at room temperature for 30 min and then washed for 2 times with TE buffer to eliminate excess amount of biotinylated oligos from the beads.

#### Ligation

The magnetic beads (product of 2.2.3) were added to a PCR tube along with 8 μL of deionized water, 2 μL of 100 μM subsequent single-stranded oligo (N1b), 10 μL of Quick Ligase Buffer, and 1 μL of T4 ligase. The PCR tube was vortexed and incubated at room temperature (25°C) for 15 min. Phosphodiester bonds were formed between the two fragments at the 5’ phosphate and 3’ hydroxyl groups.

These steps were repeated for the remainder of the oligo fragments (N2b, N3b, N4b, N5b, and N6b). Excess oligos and T4 ligase enzyme were removed by washing with 2×100 μL of TE buffer after each ligation step. At the end of the ligation process, the magnetic beads were resuspended in 20 μL deionized water.

#### PCR

The final product of the synthesis was amplified by PCR (Hybaid PCR Express HBPX gradient Thermal Cycler). The forward and reverse primers were 5’-TGCAAGCAGTGGTATCAACG-3’ and 5’-ACCATCGCCACCGTAGTC-3’, respectively. The PCR solution contained 2 μL of ligation product, 22 μL deionized water, 25 μL Emerald master mix, 1 μL DMSO and 0.5 μL of each primer. The negative control was prepared without the addition of the template DNA. The steps for the PCR program were: 95°C for 30 s, followed by 30 amplification cycles (95°C for 15 s, 65°C for 30 s, and 72°C for 20 s) and 72°C for 5 min.

#### Gel electrophoresis

PCR products were run on a 3% agarose gel in 1×TAE buffer. The horizontal electrophoresis system and power supply were obtained from Bio-Rad (Hercules, CA). The potential was set at 75 V.

#### Sanger DNA sequencing

Bands were cut from the gel, extracted, and purified using the GeneJet PCR purification kit from Fermentas (Thermo Scientific, Pittsburgh, PA). The solutions were stored in a freezer (-20°C) prior to DNA sequencing.

#### One-pot dsDNA synthesis

To perform a one-pot gene synthesis experiment, 5 μL of each of the annealed products of N1b-N2b, N3b-N4b, N5b-N6b was mixed in a tube with 10 μL Quick Ligation Buffer and 1 μL T4 Ligase. The tube was incubated at room temperature for 15 min. The final product was analyzed using gel electrophoresis.

## Results and Discussion

We have demonstrated a novel strategy to synthesize a full-length dsDNA molecule accurately and efficiently. A 101-bp long dsDNA product was synthesized by successive ligation of 28-mer fragments. The synthesis process involves annealing, binding of biotinylated oligo fragments to magnetic beads (solid support), and consecutive ligation reactions. In this study, we tested each of these steps separately to ensure the optimization of all conditions prior to synthesizing the full-length dsDNA. Oligo fragments N1b-N6b shown in Table 1 were used to test and optimize the annealing and ligation processes.

### Annealing

**Fig 3:**
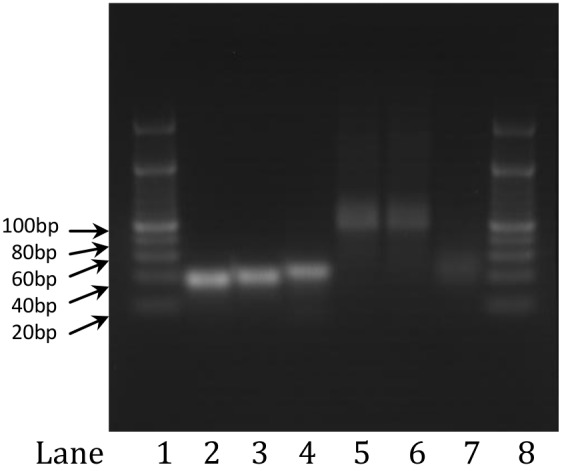
Agarose gel electrophoresis of annealed and ligation products. Lane 1 and 8, ladder; Lane 2, annealed product of N1b and N2b; Lane 3, annealed product of N3b and N4b; Lane 4, annealed product of N5b and N6b; Lane 5, “one-pot” ligation product of [(N1b-N2b)+(N3b-N4b)+(N5b-N6b)]; Lane 6, sequential ligation product of [(N1b-N2b)+(N3b-N4b)]+(N5b-N6b); Lane 7, ligation product of [(N1b-N2b)+(N5b-N6b)].

Deoxyribonucleic acids recognize their complementary oligonucleotides by base pairing: A-T and G-C. Single-stranded DNA will hybridize with its complement via the formation of hydrogen bonds. The annealing process was carried out at 95° for 10 min in an annealing buffer that promotes hydrogen bonding between the two complementary ssDNA molecules. The annealing process was tested by pairing N1b with N2b, N3b with N4b, and N5b with N6b. The annealed product was characterized using gel electrophoresis. The lengths of all the annealed products were expected to be 42 bp (28+14). Lanes 2, 3, and 4 in Fig 3 show the expected bands around 40 bp, which suggests that the annealing process was successful.

### Ligation

In this study, T4 DNA ligase was utilized as the catalyst for the formation of phosphodiester bonds between two ssDNA oligo fragments. This enzyme has been shown to effectively join both blunt and sticky ends of dsDNA. In this part of the study, we wanted to evaluate whether the T4 ligase has a favorable reaction towards dsDNA with sticky ends compared to ssDNA with blunt end. T4 ligase is most commonly used to join dsDNA fragments. In our approach we used this enzyme to join ssDNA fragments with the complementary sticky ends of dsDNA molecules (Fig 1).

To better understand the efficiency of the T4 ligase in joining our dsDNA fragments with sticky ends, we first performed a sequential ligation reaction using the annealed products of N1b-N2b, N3b-N4b, and N5b-N6b described in section 3.1. The first ligation was carried out between the N1b-N2b and N3b-N4b dsDNA fragments and was allowed to incubate at room temperature for 15 min. The next dsDNA fragment, N5b-N6b, was then added into the solution mixture and incubated under the same conditions as the first ligation. The resulting approximately 100 bp product is shown in Fig 3, Lane 6, and is consistent with our expected product size of 98 bp (28+28+28+14 bp). This confirms that the sequential ligation of dsDNA fragments with complementary sequences can be accomplished at room temperature in as fast as 15 minutes.

We also investigated whether or not the T4 ligase enzyme was able to effectively join dsDNA fragments at their complementary sites in the presence of impurities in the form of other dsDNA fragments. A “one-pot” reaction was performed by mixing all of the annealed products with the T4 ligase in a tube, which was incubated at room temperature (25°C) for 15 min. The resulting product was run on a gel and showed a similar band at ~100 bp (Fig 3, Lane 5). To determine whether or not ligation occurs between the N1b-N2b and N5b-N6b fragments, we ran a similar test in which only these two dsDNA fragments were mixed with the T4 ligase and incubated at 25°C for 15 min. The product was again analyzed on a gel and the result showed a band around 40 bp that corresponded to the sizes of the annealed products themselves (42 bp expected) and not the product of successful ligation (Fig 3, Lane 7). These two annealed products were not designed with complementary sequences in their overhang portions. This result indicated that ligation of dsDNA fragments with non-complementary sticky ends was unfavorable under the specified reaction conditions with T4 ligase. This feature plays an important role in the proposed gene synthesis approach, as it shows that ligation is only favorable in the presence of DNA fragments with complementary sticky ends. This significantly reduced the chance of misalignments or sequence errors in the final gene product as a result of non-complementary oligo fragments that may be present in solution during the ligation process.

### Oligonucleotide synthesis using streptavidin-coated magnetic beads

The proposed gene synthesis strategy began with the annealing process, where the biotinylated A1B fragment was annealed with the A2b fragment to form the initial dsDNA molecule. The annealed product of A1B/A2b was immobilized on magnetic beads via streptavidin-biotin binding. Magnetic beads allow for convenient washing and separation of the desired products from excess reagents. Holmberg et al.[18] demonstrated that dissociation of biotin and streptavidin molecules occurs when a solution is heated to 70°C. Thus, the annealing of the first dsDNA fragment was performed prior to its immobilization on the magnetic beads to avoid exposing the immobilized products to the elevated temperature of the annealing process (95°C).

**Fig 4:**
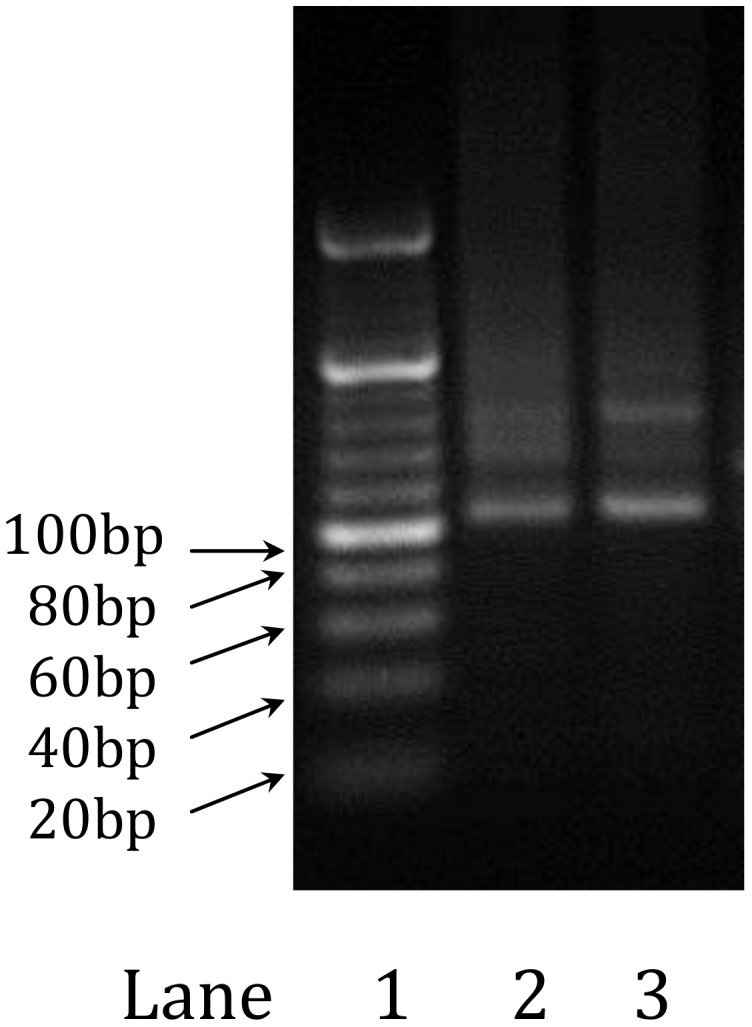
Agarose gel electrophoresis of the final product. Lane 1, ladder; Lane 2, ligation product from double-stranded oligo fragments; Lane3, ligation product from single-stranded oligo fragments.

The results from section 3.2 demonstrated that T4 DNA ligase was capable of joining dsDNA fragments with complementary sticky ends. In this experiment, the ligation process was repeated by adding the annealed products of N1b-N2b, N3b-N4b and N5b-N6b one at a time to the main chain. At the completion of the ligation reactions, the full-length dsDNA was amplified by PCR. The result is illustrated in Fig 4, lane 2, as a band of approximately 100 bp, which corresponds to the expected length of the final product (101 bp).

We also investigated whether or not it was possible to ligate single-stranded oligonucleotides sequentially to the main dsDNA chain using the T4 DNA ligase under the same experimental conditions described in section 3.2. To test this, 28-mer ssDNA fragments were used as building blocks (rather than the double-stranded oligos), and were ligated one at a time to extend the length of the main chain. Thorough washing was performed at the completion of each ligation step to minimize interference from excess oligos that may still be present in solution. Once all of the ligation steps were completed to form the desired dsDNA, the product was amplified by PCR and analyzed using gel electrophoresis. The result of the PCR product is illustrated in Fig 4 lane 3 and clearly shows a product of approximately 100 bp. We have successfully demonstrated an efficient approach to synthesizing and extending the length of dsDNA through repetitive ligation of short ssDNA oligo fragments. Each cycle of the DNA synthesis step takes approximately 20 minutes, including the cleaning and washing steps. Our proposed method would take only about 12 hours to synthesize a DNA molecule of 500 bp, whereas today’s commercial gene synthesis operations generally require multiple days to synthesize DNA molecules of this size.

### Sequencing of the target DNA

PCR products were cut from the agarose gels, extracted, and purified using the GeneJet PCR purification kit (ThermoScientific) for sequencing analysis. Sanger sequencing was performed to characterize the final products. Since the quality of Sanger sequencing was normally poor for the first 20 bp of the DNA sequence, sequencing of the DNA products was performed in both the forward and reverse directions to obtain the whole sequence data. The reproducibility of the gene synthesis approach and the accuracy of the DNA sequencing results were determined by repeating the entire process in triplicate. The resulting PCR product for each process was sequenced 3 times. Sequencing data were aligned and compared to the expected sequence by CodonCode Aligner (v. 5.1.5 CodonCode Corp., MA). The sequence validation by Sanger sequencing revealed that gene synthesis product was nearly identical (97%) to the expected sequence. DNA sequencing alignment is shown in Supporting Information.

## Conclusions

In this work, we have demonstrated a novel approach of synthesizing a long double stranded oligonucleotide through repetitive ligation of short ssDNA oligo fragments. The method is simple and straightforward, using streptavidin coated magnetic beads as solid supports for ease of washing. The magnetic bead purification for each ligation-based assembly helps promote the assembly of a pure and accurate dsDNA product and enables complete automation of the synthetic approach–work that is underway in our lab. The accuracy of the synthesis method was validated by Sanger sequencing, and the results showed we were able to generate DNA products with precision of more than 97%. With this approach, it is possible to create custom DNA with rapid turnaround time. Combined with automation technology and access to universal libraries of short oligo fragments, this approach would provide a powerful gene synthesis solution with significant time and cost savings.

## Supporting Information

S1 Fig DNA sequencing alignment of the final product. The data obtained via Sanger sequencing was aligned using CodonCode Aligner (v. 5.1.5 CodonCode Corp., MA). This figure illustrates the quality of the actual product relative to the intended product. Errors are indicated by the blue arrows.
(TIF)
