# Supplementary figures and images for: Rapid Synthesis of a Long Double-Stranded Oligonucleotide from a Single-Stranded Nucleotide Using Magnetic Beads and an Oligo Library
Source: PLoS One. 2016 Mar 1;11(3):e0149774. doi: 10.1371/journal.pone.0149774 (PMC4773231; doi:10.1371/journal.pone.0149774)

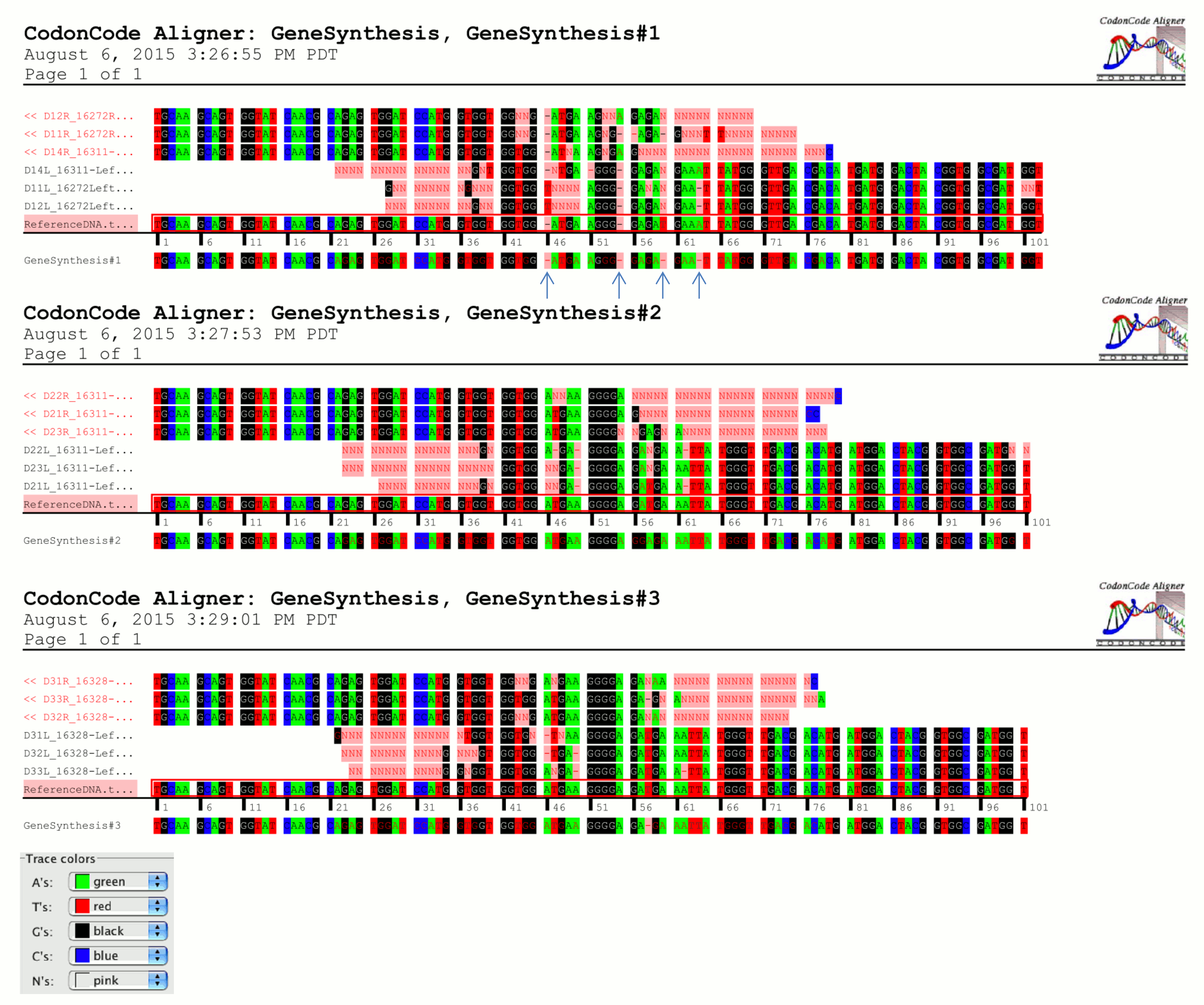

Supplement: S1 Fig — The data obtained via Sanger sequencing was aligned using CodonCode Aligner (v. 5.1.5 CodonCode Corp., MA). This figure illustrates the quality of the actual product relative to the intended product. Errors are indicated by the blue arrows. (TIF) [file pone.0149774.s001.tif]
